# Syrian Refugee Youth Resettled in Norway: Mechanisms of Resilience Influencing Health-Related Quality of Life and Mental Distress

**DOI:** 10.3389/fpubh.2021.711451

**Published:** 2021-09-23

**Authors:** Cecilie Dangmann, Øivind Solberg, Anne Kjersti Myhrene Steffenak, Sevald Høye, Per Normann Andersen

**Affiliations:** ^1^Faculty of Health and Social Sciences, Inland Norway University of Applied Sciences, Elverum, Norway; ^2^Department of Health Science, Swedish Red Cross University College, Stockholm, Sweden; ^3^Department of Psychology, Inland Norway University of Applied Sciences, Lillehammer, Norway

**Keywords:** health-related quality of life, mental distress, post-migration stressors, post-traumatic stress disorder, refugee, resilience, Syria, youth

## Abstract

**Background:** The importance of resilience factors in the positive adaptation of refugee youth is widely recognised. However, their actual mechanism of impact remains under-researched. The aim of this study was therefore to explore protective and promotive resilience mechanisms to inform future interventions and policy. Promotive resilience is seen as a direct main effect and protective resilience as a moderating effect.

**Methods:** This was a cross-sectional study of Syrian youth recently resettled in Norway, aged 13–24 years. Regression and moderation analyses were used to explore different interactions, including moderated mediation using the PROCESS macro for SPSS.

**Result:** A direct main effect of promotive resilience was found for health-related quality of life (HRQoL) and general mental distress, but not for post-traumatic stress disorder (PTSD). No moderating effects of protective resilience were found. Post-migration stressors mediated the effects of potentially traumatic events (PTE) from war and flight, and this indirect effect was present at all levels of resilience.

**Conclusion:** Despite high risk exposure and mental distress, resilience was also high. Broad resilience interventions targeting multiple factors would likely benefit the group, but these should include both individual assets and building supportive environments. Additionally, reducing current stress and providing treatment for those in need could enable recovery and increase the efficacy of resilience factors already present.

## Introduction

The refugee experience of war, violence, and forced migration is associated with negative impacts on the mental health of children and youth, lasting well into their resettlement (1, 2). Whilst it is important to understand and address refugees' mental health problems, their capacity for resilience must be respected (3). Despite the potentially traumatic events (PTEs) caused by warfare, studies suggest that resilience is the norm. Findings indicate that the majority of refugees retain or achieve positive health and well-being during the resettlement process (4–6). Resilience factors—such as social support or access to services—are generally associated with better mental health in displaced populations (7, 8). However, the underlying mechanisms remain under-researched; as such, resilience processes central to mental health in refugee youth may be overlooked (9, 10). This knowledge is important for protecting and promoting the individual and environmental resources necessary for positive adaptation.

The framework of resilience can be used to answer the question of why some children and youth adapt whilst others develop problems in response to stress and trauma. Several conceptions of resilience exist, but in this study we refer to the socio-ecological and cross-culturally relevant definition of resilience proposed by Ungar: “In the context of exposure to significant adversity, resilience is both the capacity of individuals to navigate their way to the psychological, social, cultural, and physical resources that sustain their wellbeing, and their capacity individually and collectively to negotiate for these resources to be provided in culturally meaningful ways” (11).

Socio-ecological explanations define resilience as a process that is co-facilitated by individuals and their physical and social ecologies: this contrasts an exclusive focus on individual factors (12). Supporting this, reviews of resilience in refugee children and youth reveal a plethora of interlinked factors on several levels. At an individual level, self-regulation, coping mechanisms, and self-efficacy have shown protective effects (13, 14). Family and parental factors are of utmost importance for refugee children and youth; these emphasise how the family may function as a key emotional regulator, buffering, or exacerbating the impact of earlier PTEs from war and flight (10, 15–17). Family cohesion, perceived parental support, and parental mental health—particularly in mothers—are associated with fewer psychological difficulties in children (7). Friends and the subjective experience of peer relationships are also integral to healthy psychological development in children and youth (7, 17, 18). Friendships may prevent social isolation and loneliness and infer a sense of belonging, especially in school (19). Perceptions of acceptance and belonging within schools and wider communities are linked to self-esteem, identity development, and acculturational processes (6, 10). It is also suggested that cultural identity and specific competencies—such as language skills—play an important role for well-being in resettlement (4, 16).

However, the above resilience factors and processes are often investigated separately and scattered throughout several contexts, despite the acknowledgement of complex interactions (9). Evidence supports a multiple factor model where the total constellation of resilience factors promotes better functioning after adversity, not one specific driving factor (7, 10, 17, 20). A composite measure was therefore included in this study. The Child and Youth Resilience Measure (CYRM) is cross-culturally developed (21) and includes resilience factors also identified as important for refugee children and youth (22).

Although several resilience factors are known, *how* they influence mental health is less explored. Resilience mechanisms are the theoretical and operational process by which the outcome is thought to be achieved (23). Two of the more influential models are *promotive* and *protective resilience* models (24–27). *Promotive resilience* models are also termed “compensatory” or “additive,” suggesting that positive resilience factors compensate for the presence of negative risk. The mechanism is manifest when a resilience factor has a direct influence on the outcome (i.e., a main effect in the analysis) and does not interact with a risk factor in predicting the outcome. Thus, the factor influences all participants in the same manner, both those exposed and not exposed to the risk. On the other hand, a *protective resilience* model proposes that factors buffer the influence of risk, identified when the resilience factor interacts with the risk factor to predict the outcome. Thus, the protective resilience factor is especially influential when risk is present. A protective effect would imply that for someone with low resilience, experiencing trauma or stress would lead to a steep increase in mental distress. By contrast, experiencing similar trauma or stress for someone with high resilience would lead to a much smaller increase in mental distress—or no increase at all.

When exploring resilience, it is also important to consider the relevant outcomes and risk exposure (28). Mental distress, such as post-traumatic stress disorder (PTSD), anxiety, and depression, are frequently used as outcomes in refugee studies (1, 7). However, seeing mental health as more than the absence of mental distress requires broader and multidimensional outcomes, and these are under-researched in this population (29). Three outcomes were therefore examined in this study: (1) general mental distress (symptoms of depression and anxiety), (2) PTSD (symptoms of intrusion and avoidance), and global health-related quality of life (HRQoL) (a quality of life index related to multiple dimensions of health and well-being). It is suggested that, since quality of life and mental distress are different concepts and not opposite ends of a scale, different resilience mechanisms may be relevant in reducing mental distress or increasing quality of life (10); as such, all outcomes were analysed separately.

With regards to relevant risk exposure for refugee children and youth, experiences from war and flight—such as violence, seeing someone die, or fearing for one's life—are commonly reported among refugee children, including Syrian youth (30). These PTEs are repeatedly associated with negative outcomes, such as increased mental distress (7, 8) and reduced quality of life (29, 31). In addition, studies suggest that risk factors after settlement—*post-migration stressors—*may impact health and wellbeing above and beyond those of PTEs, even more prominently so in children and youth (32, 33). Stressors such as uncertain legal status, language difficulties, economic concerns, and discrimination are shown to directly increase mental distress in refugees, including Syrian youth (30).

In their *ecological model of refugee distress*, Miller and Rasmussen proposed a mediational effect where PTEs increase the amount of experienced post-migrations stressor (34). Later studies have found empirical support for this model (33, 35–37). One proposed mechanism is that trauma sensitises a person to future adversity, triggering an overreaction to ongoing demands (5, 38). To our knowledge, few have explored if this indirect effect of post-migration stressors is affected by resilience.

**Figure 1:**
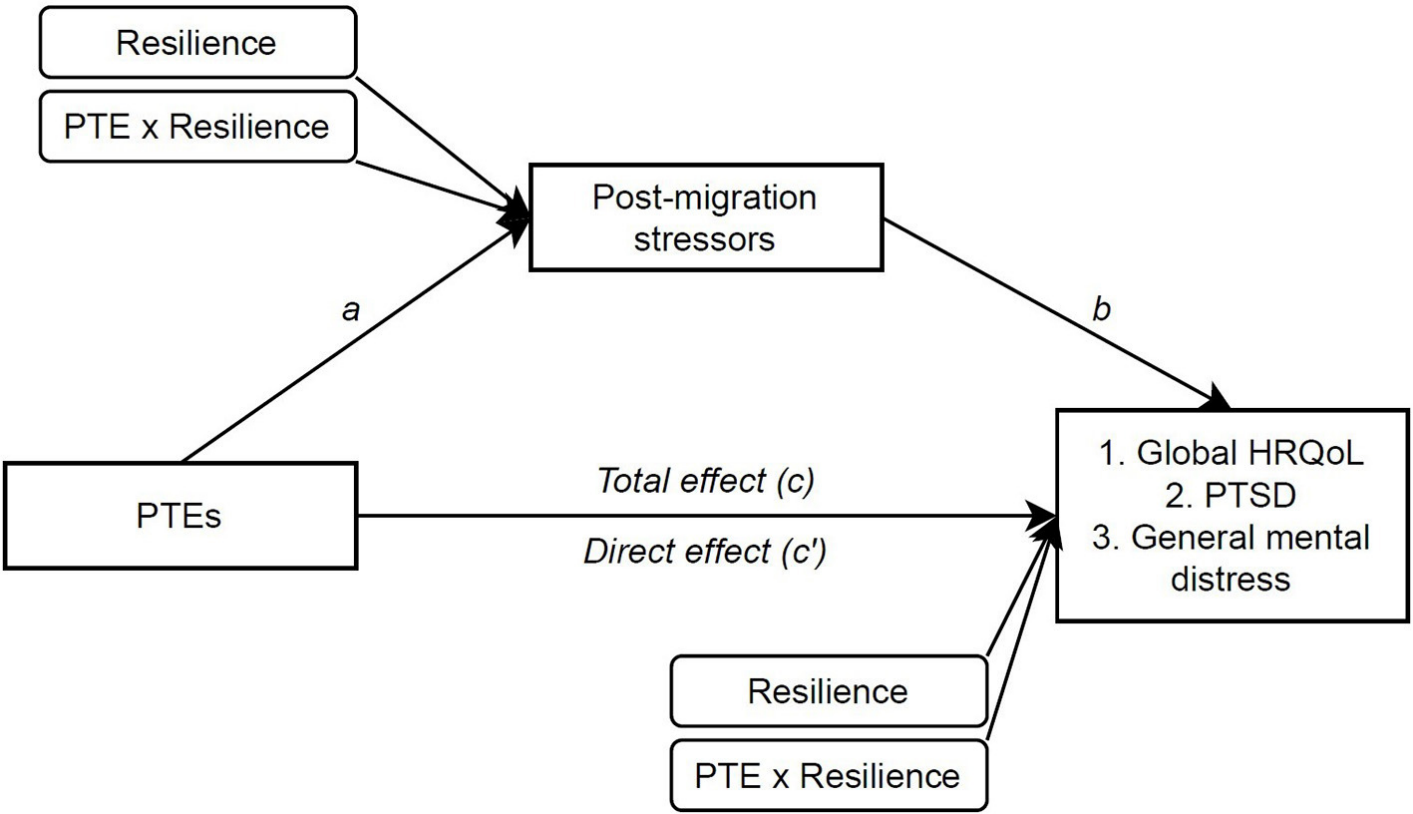
Conceptual model of moderated mediation analyses, SPSS PROCESS model 8. Paths *a* represent the relationship between the predictor and the mediator, and path *b* between mediator and outcome whilst the predictor value is controlled. The *c*′ path is the direct effect between the predictor and the outcome excluding the mediator variables. The indirect effect is indicated by a statistically significant difference between *c* and *c*′. The indirect effect would be significant if CIs do not include zero. “Resilience” indicates the direct main effect (promotive resilience), and “PTE × Resilience” indicates the interaction term (protective resilience).

The aim of this study was to analyse the main and moderating effect of resilience on mental distress and quality of life outcomes, related to risk factors from both pre- and post-settlement. Firstly, we hypothesised that resilience has a direct main effect on all the outcomes, suggesting *promotive resilience*. Secondly, we hypothesised that resilience buffers the negative effects of all risk factors on all outcomes, suggesting *protective resilience*. Our third hypothesis was two-fold: (1) that post-migration stressors mediate the association between PTEs and all outcomes; and (2) that resilience moderates the relationship between pre-and post-migration risks and the potential mediation (see Figure 1).

## Materials and Methods

### Design and Setting

This study forms part of a larger research project “Good starts—mental health and resilience in Syrian refugee youth” with two previously published articles including the same study sample (31, 37). The present study utilised a cross-sectional, questionnaire-based design. Questionnaires were administered in both Arabic and Norwegian. The teachers distributed written information about the study in Arabic and Norwegian to the students in advance and consent forms to parents with children under the age of 16. The youth consenting to participate completed the questionnaire whilst at school, with a researcher present to answer questions.

### Participants

The inclusion criterion for age was 12–24 years, as refugees in Norway have the right to attend secondary and upper secondary schools. Using strategic sampling to recruit recently resettled youth, 40 schools with introductory classes for newly arrived immigrants were contacted. Twenty-three schools located in nine different regions of Norway agreed to participate. Reasons for not participating were *no response to request* (nine schools), *no Syrian students* (six schools), or *already participating in other studies* (two schools). The participating schools had between 1 and 23 Syrian students in attendance. Three students declined to participate due to exam preparations or language difficulties. The recruitment period lasted from May to December 2018, and a total of 160 youth from Syria were included in the final sample. For further details concerning methods and materials, see also (31, 37).

### Measures

All measures had validated Arabic language versions available, and permission to use these were sought from all copyright holders. Measures for PTE and post-migration stressors were translated from Swedish to Norwegian and reviewed to detect and remove any discrepancies in meaning. A cultural expert reviewed the questionnaire for cultural appropriateness and comprehensibility, and a pilot study was conducted in a refugee learning centre.

#### Potentially Traumatic Events

The Refugee Trauma History Checklist was developed for self-report data on refugee trauma history in community samples, considering intrusiveness, and relevance of the included events (39). Adjustments to fit local context and group are advised, the measure was therefore modified to fit the age of respondents and the context of recent resettlement e.g., by reducing the number of items in the scale. The list consisted of 10 dichotomous (yes/no) items: witnessing war; being forced to leave friends/family; having someone you love disappear; experiencing someone trying to hurt you or someone you love; having a life-threatening illness or injury; lacking food or shelter; having to hide; being tortured; seeing someone die; or feeling as though your life was in danger. All positive responses were added as a cumulative score (range 0–10), with higher scores indicating a higher number of experienced events.

#### Post-migration Stressors

Post-Migration Stress Scale (RPMS) is an instrument for assessing post-migration stressors, validated in Syrian refugees in Sweden (40). This was modified to fit the age of the respondents and the Norwegian context, e.g., by reducing the number of items and rewording to fit a Norwegian context. Ten indicators were used, representing different types of stressors experienced since the respondents arrival in Norway: perceived discrimination, language difficulties, economic strain, missing family, family cultural conflicts, feeling lonely, missing their previous life, feeling unsafe, worrying about having to move, or worrying about having to leave Norway. All indicators were scored on a five-point Likert scale, ranging from 0 (never) to 4 (very often) and were added as a cumulative score. Higher scores indicate higher frequencies of experienced stressors (range 0–40), and the Cronbach's alpha was 0.77 in this study.

#### General Mental Distress

The Hopkins Symptom Checklist (HSCL-10) consists of four items related to anxiety and six related to depression that collectively indicate general mental distress (41). All items have four response categories ranging from 1 (not at all) to 4 (extremely), regarding how much the symptoms bothered the respondents over the past 7 days. The response values are added together and then divided by the number of items (range 1–4): higher scores indicate greater symptom load. A cut-off score of ≥1.85 was used as an indication of general mental distress (41). The HSCL-10 has previously shown satisfactory validity and reliability as a measure of mental distress in both young and displaced populations (42). The Cronbach's alpha in this study was 0.89.

#### Post-traumatic Stress Disorder

The Child Revised Impact of Events Scale (CRIES-8) is a screening tool measuring the severity of post-trauma intrusion and avoidance symptoms over the past week. Eight items are rated on a four-point scale ranging from 0 (not at all), 1, 3–5 (often); they are then added together (range 0–40), with higher scores indicating greater symptom loads. The scale is recommended by the Children of War Foundation and has been cross-culturally validated with good psychometric properties, with a cut-off value of ≥17 indicating possible PTSD (43). The Cronbach's alpha in this study was 0.86.

#### Global Health-Related Quality of Life

Health-Related Quality of Life is a multidimensional construct considered to represent the health aspect of quality of life, focusing on people's daily functioning and ability to experience a fulfilling life (44). KIDSCREEN-10 is a self-report measure of global HRQoL developed through Rasch analysis, ensuring that only items that represent a global, unidimensional latent trait are included (45). It has been cross-culturally validated in 38 languages and includes elements from physical and psychological well-being, relationships, autonomy, and school environment. The items are rated on a scale from 1 to 5 for experiences in the past week; a scoring algorithm was used to calculate T-scores with a mean of 50 and a standard deviation of 10, with higher scores indicating higher self-rated HRQoL (45). Permission was obtained from the KIDSCREEN organisation, and the Arabic and Norwegian versions were downloaded from their member web pages. The Cronbach's alpha in this study was 0.82.

#### Resilience

The CYRM is based on a socio-ecological framework and assesses resilience factors within individual, relational, and contextual dimensions. It was cross-culturally developed and validated through the gathering of data from youth in 11 western and non-western countries (21, 46). The CYRM-12 is a brief measure derived from the original 28 items and including items from all dimensions (47). The items are rated on a scale from 1 to 5 (range 12–60) regarding experiences in the last week, where higher scores indicate higher resilience. The Arabic version of this measure was translated and validated for Syrian refugee youth resettled in Jordan (22). The measure was then reviewed by Syrian youth living in Norway and teachers in introductory classes before it was piloted. The Cronbach's alpha in this study was 0.79.

### Statistical Analyses

General descriptive statistics, *t*-tests, and correlation analyses were used to explore the variables. In multiple regressions, the variables were entered sequentially in multiple regressions to examine the main effect of resilience after controlling for risk and interaction effects (hypothesis 1). Interaction effects between both risks and resilience was also evaluated in the same regression (hypothesis 2). Mediation, and any moderating effect on this mediation (hypothesis 3) were analysed in in a regression- based approach using 5,000 bootstrap samples in the SPSS PROCESS 3.4 macro (models 4 and 8), in a procedure suggested by Hayes (48). The models assess possible mediation or indirect effects, the moderation of the relationship between the predictor and mediator (*a*), the residual direct effect (*c*′), and the total indirect effect (*ab*) (see Figure 1). An indirect or moderation effect is assumed to be significant at an alpha level of 0.05 if its 95% confidence interval (CI) does not include zero. Gender and age were controlled for as covariates, when relevant. To further explore the multidimensional aspect of resilience, a *post-hoc* correlational analysis of each resilience factor was done. Missing analyses for all measures showed that all items were missing at random. Any participant with two or more missing items from the measures were excluded from analyses, including 19 participants who opted for a shortened questionnaire due to language difficulties, which did not contain HSCL-10, CRIES-8, or post-migration stressors. Remaining missing items (<2%) were replaced by methods advised in separate instrument manuals, such as the KIDSCREEN handbook (45). Central assumptions of linearity, homoscedasticity, normality, and multicollinearity were tested and no violations of these assumptions were detected (49). Also, no significant bias due to imputed values was observed.

## Results

### Sample Characteristics

The sample included 160 youth from Syria (37.5% female, mean age 18 years) (Table 1). All participants attended school full-time in either local secondary or upper secondary schools or adult learning centres. The average time since they left Syria was 5.3 years, and their mean time in Norway was 2 years. Most had Arabic as their mother tongue (76.3%). The majority were living with their parents (75%); of those who were not, most were >19 years of age (M_age_ = 20.1 years, range 16–24 years), lived alone and were male (89%).

**Table 1:** Sociodemographics of the participants (*n* = 160).

**Descriptives**	**Mean (SD)**	**Range**	***n* (%)**
Gender			
*Female*			60 (37.5)
*Male*			100 (62.5)
Age	18.1 (2.4)	13–24	
Years as refugee	5.3 (1.9)	0–10	
Years of residence in Norway	2.0 (1.2)	0–8	
No. of moves past 5 years	3.7 (2.9)	1–15	
Mother tongue			
*Arabic*			121 (76.3)
*Kurdish*			34 (21.9)
*Other*			3 (1.8)
Living with parents			
*Yes*			120 (75)
*No*			35 (21.9)
*Unknown*			5 (3.1)

### Descriptives and Correlations Between Study Variables

The means and correlation coefficients are presented in Table 2. The participants (*n* = 145) reported a mean of 4.6 PTEs (*SD* = 2.61), the most prevalent being *witnessing war* (68%), *feeling your life was in danger* (59%), and *seeing someone die* (55%). A total of 88% reported at least one event, and 61% four or more events. Youth older than 18 years had experienced significantly more PTEs [19–24 years: *t*_(145)_ = −3.36, *p* = 0.001] and boys more so than girls [Boys: *t*_(145)_ = −3.38, *p* = 0.001]. Scores for post-migration stressors were an average of 13.48 (*SD* = 7.54) with no gender differences, but again the youth over 18 years reported more frequent stressors [*t*_(122)_ = −2.35, *p* = 0.020]. The mean score for general mental distress (HSCL-10) (*n* = 127) was 1.77 (*SD* = 0.61), and 36% had scores above the recommended threshold (≥1.85) considered an indication of mental distress (35). Almost half the participants (48%) had scores above the recommended threshold for PTSD (≥17) (37). The mean score for global HRQoL (44.52, *SD* = 10.05) was lower than norm data (48.51, *SD* = 9.28) and 26% reported low HRQoL (39). The resilience mean score was 47.11 (*SD* = 8.10), and 49% had scores above the median (47.0). Although age and resilience were correlated, there was no significant difference in resilience between age groups (13–18 vs. 19–24 years).

**Table 2:** Correlations between main study variables.

	**Mean (SD)**	**Min–max**	**1**	**2**	**3**	**4**	**5**	**6**
1. Global HRQoL	44.52 (10.05)	18.5–83.8	–					
2. PTSD	17.20 (10.17)	0–38	–0.34^***^	–				
3. General mental distress	1.77 (0.61)	1–3.8	–0.60^***^	0.39^***^	–			
4. PTEs	4.56 (2.61)	0–10	–0.30^***^	0.55^***^	0.27^**^	–		
5. Post–migration stressors	13.48 (7.54)	0–33	–0.45^***^	0.48^***^	0.33^***^	0.55^***^	–	
6. Resilience	47.11 (8.10)	20–60	0.61^***^	−0.23^*^	–0.45^***^	–0.20^*^	–0.27^**^	–
7. Age	18.12 (2.41)	13–24	–0.32^***^	0.11	0.14	0.38^***^	0.27^**^	–0.23^**^
8. Gender			0.02	0.004	−0.13	0.29^***^	0.15	−0.09

*Outcomes: HRQoL, health-related quality of life (KIDSCREEN-10); PTSD, post-traumatic stress disorder (CRIES-8); general mental distress (HSCL-10); Risks: PTE, potentially traumatic events; RTHC, post-migration stressors (RPMSS); Resilience (CYRM-12). Covariates: Age (continuous) and gender (female = 0). Spearman's rho was used in analyses due to skewness in some variables. Significant values after Bonferroni–Holmes correction are shown in bold.*

* *p < 0.05*,

** *p < 0.01*,

*** *p < 0.001 (two-tailed)*.

Resilience was positively correlated with HRQoL and negatively correlated with general mental distress and PTSD, whilst risk factors (PTEs and post-migration stressors) were negatively correlated with HRQoL and positively correlated with PTSD and general mental distress. No correlations between the predictors exceeded the 0.80 multicollinearity threshold suggested by Field (50).

### Promotive and Protective Resilience Mechanisms

Variables were entered sequentially in multiple regressions for the three separate outcomes: HRQoL, PTSD, and general mental distress. Age and gender were entered first as covariates, and the risk factors (PTEs and post-migration stressors) second. Resilience was entered separately in the third model to assess its individual contribution to each outcome (main effect or *promotive resilience*). The interaction term between risk factors and resilience were entered last, to assess possible moderation as suggested in hypothesis 2 (*protective resilience*) (see Table 3).

**Table 3:** Multiple regression for resilience effects on HRQoL, PTSD, and general mental distress.

		**HRQoL**			**PTSD**			**General mental distress**		
		***b***	***p***	**Δ*R*^**2**^**	***b***	***p***	**Δ*R*^**2**^**	***b***	***p***	**Δ*R*^**2**^**
Model 1										
	Age	−10.26	<0.001^***^		0.66	0.138		0.05	0.050^*^	
	Gender	10.53	0.322		−0.21	0.917		−0.27	0.030^*^	
	Δ*R*^2^			0.114^***^			0.020			0.056^*^
Model 2										
	Age	−0.80	0.014^*^		−0.22	0.548		0.03	0.342	
	Gender	10.98	0.180		−20.30	0.163		−0.33	0.005^**^	
	PTE	−0.38	0.252		10.91	<0.001^***^		0.05	0.079	
	Post-stress	−0.33	0.003^**^		0.40	0.001^***^		0.02	0.014^*^	
	Δ*R*^2^			0.124^***^			0.384^***^			0.132^***^
Model 3										
	Age	−0.62	0.027		−0.23	0.537		0.01	0.641	
	Gender	10.80	0.153		−20.29	0.167		−0.31	0.004^**^	
	PTE	−0.25	0.380		10.90	<0.001^***^		0.04	0.132	
	Post-stress	−0.24	0.011^*^		0.39	0.002^**^		0.02	0.047^*^	
	Resilience	0.53	<0.001^***^		−0.04	0.727		−0.03	<0.001^***^	
	Δ*R*^2^			0.215^***^			0.001			0.128^***^
Model 4										
	Age	−0.64	0.025^*^		−0.25	0.501		0.01	0.634	
	Gender	10.93	0.128		−20.29	0.172		−0.31	0.005^**^	
	PTEs	−0.29	0.311		10.93	<0.001^***^		0.031	0.201	
	Post-stress	−0.24	0.012^**^		0.39	0.002^**^		0.02	0.046^*^	
	Resilience	0.54	<0.001^***^		−0.06	0.619		−0.03	<0.001^***^	
	PTE x Resilience	−0.02	0.612		0.030	0.520		−0.01	0.384	
	Post-stress x Resilience	−0.01	0.520		−0.009	0.573		0.00	0.922	
	Δ*R*^2^			0.005			0.003			0.006
Final model *R*^2^			0.458^***^			0.408^***^			0.32^***^

*Outcomes: HRQoL, health-related quality of life (KIDSCREEN-10); PTSD, post-traumatic stress disorder (CRIES-8); general mental distress (HSCL-10). Risks: PTE, potentially traumatic events (RTHC); post-stress, post-migration stressors (RPMSS). Moderator: resilience (CYRM-12). Covariates: age (continuous) and gender (female = 0). Interaction variables are all centred.*

* *p < 0.05*,

** *p < 0.01*,

*** *p < 0.001*.

Of the two risk factors, only post-migration stressors had a significant main effect on HRQoL, contributing to 12.4% of the variance [*R*^2^ change = 0.12, *F* change_(2, 115)_ = 9.33, *p* < 0.001]. However, resilience contributed to almost double that amount individually [*R*^2^ change = 0.22, *F* change_(1, 114)_ = 44.77, *p* < 0.001]. The interaction terms were not significant. The final model was significant and explained 46% of the total variance in HRQoL [*Adjusted R*^2^ = 0.42, *F*_(7, 112)_ = 13.52, *p* < 0.001]. For PTSD as an outcome, PTE and post-migrations stressors were the only significant contributors explaining 38% of the variance [*R*^2^ change = 0.38, *F* change_(2, 110)_ = 35.44, *p* < 0.001]. The final model explained about 41% of the variance in PTSD symptoms [*Adjusted R*^2^ = 0.37, *F*_(7, 107)_ = 10.52, *p* < 0.001]. Lastly, for general mental distress as an outcome, only post-migration stressors contributed significantly to model 2, and risk factors explained 13% of the variance [*R*^2^ change = 0.13, *F* change_(2, 112)_ = 9.11, *p* < 0.001]. Introducing resilience in model 3 significantly increased the explained variance with another 13% [*R*^2^ change = 0.13, *F* change_(1, 111)_ = 20.74, *p* < 0.001]. The interaction terms were not significant, and the final model explained a total of 32% of the variance in mental distress [*Adjusted R*^2^ = 0.28, *F*_(7, 109)_ = 7.40, *p* < 0.001].

**Figure 2:**
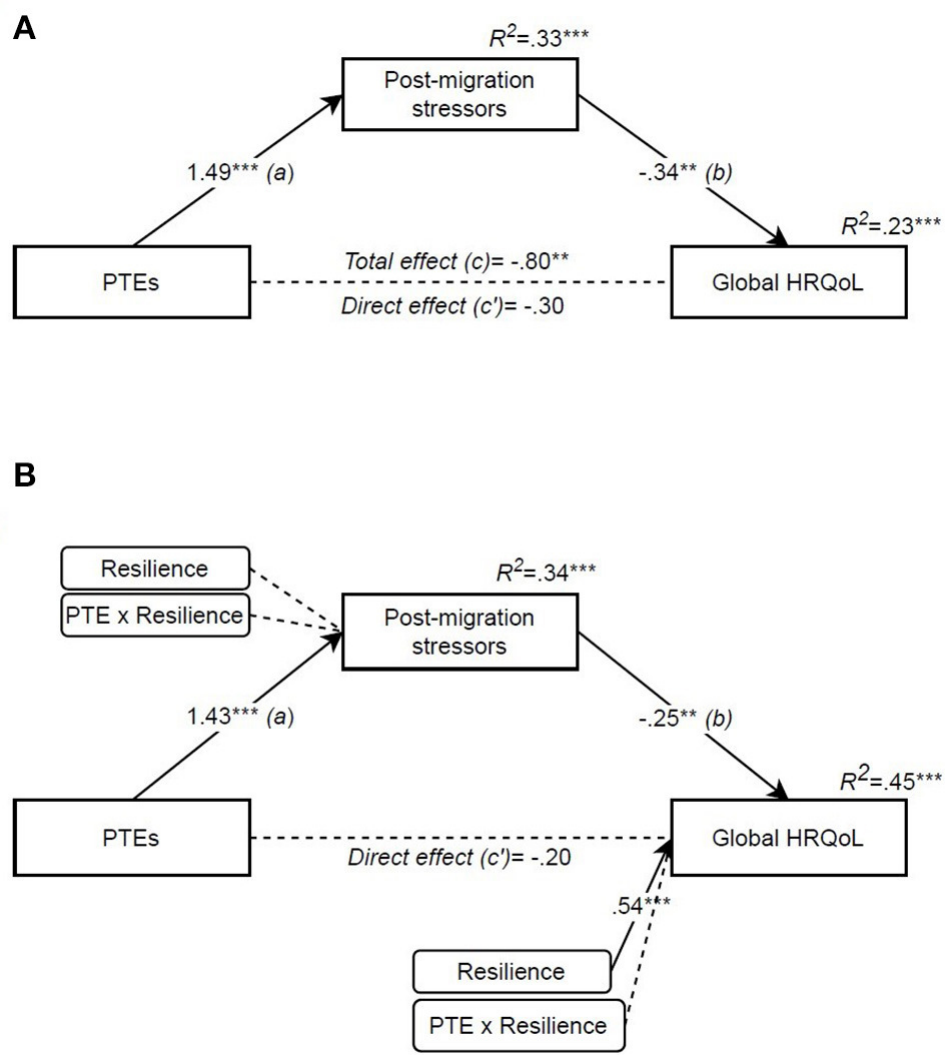
Potentially traumatic events' effect on Global HRQoL mediated by post-migration stressors and moderated by resilience. **(A)** Mediation (PROCESS model 4), **(B)** Moderated mediation (PROCESS model 8). Independent variable: PTE [potentially traumatic events (RTHC)]. Mediator: post-migration stressors (RPMSS). Outcome: global HRQoL [health-related quality of life (KIDSCREEN-10)]. Moderator: resilience (CYRM-12). Covariate: age. All coefficients are unstandardized. *p < 0.05, **p < 0.01, ***p < 0.001.

To explore the last hypothesis, PTE and post-migrations stressors were analysed in mediational analyses for each outcome (PROCESS model 4). Moderation effects of resilience (protective resilience) were entered on paths *a* and *c*′ *(PROCESS model 8)* (see Figure 1). Considering the results of the final regression models, only age was included as a covariate in the model for HRQoL and gender for general mental distress, to maintain the highest possible power of the estimates. The results for HRQoL as the outcome are presented in Figure 2.

The mediation analysis supported our third hypothesis (Figure 2A), showing that PTEs influenced quality of life indirectly through post-migration stressors, and that this indirect effect was significant [*indirect effect* (*a* and *b*): −0.50, 95% CI (−0.86, −0.23)]. The model accounted for 23% of the variance in HRQoL scores [*F*_(3, 116)_ = 11.27, *p* < 0.001, *R*^2^ = 0.226], where age contributed to about half of this variance (see also Table 2). Adding resilience as a moderator (Figure 2B) did not result in significant interaction in the first stage of the model (*a*): resilience therefore did not moderate PTEs' influence on the amount of experienced post-migration stressors, nor contribute significantly to the amount of post-migration stressors. The residual direct effect between PTEs and HRQoL (*c*′) was not moderated by resilience, however resilience scores contributed directly to the variance in HRQoL [total model: *F*_(5, 114)_ = 18.30, *p* < 0.001, *R*^2^ = 0.445]. Lastly, the indirect effect of PTEs through an increase in post-migration stressors was significant at all levels of resilience (see Table 4), indicating no moderation of the indirect effect.

**Table 4:** Conditional indirect effects of PTEs on HRQoL, PTSD, and general mental distress.

**Level of resilience**	**Effect**	**Boot CI**	**Boot CI**
**Health-Related Quality of Life**			
−1 SD	−0.34	−0.73	−0.10
Mean	−0.35	−0.64	−0.012
+1 SD	−0.37	−0.66	−0.10
Index of moderated mediation	−0.002	−0.02	0.03
**Post-Traumatic Stress Disorder**			
−1 SD	1.66	0.18	1.14
Mean	1.77	0.21	1.04
+1 SD	1.87	0.16	1.12
Index of moderated mediation	0.004	−0.04	0.03
**General Mental Distress**			
−1 SD	0.024	0.001	0.056
Mean	0.025	0.001	0.054
+1 SD	0.026	0.001	0.061
Index of moderated mediation	0.0008	−0.002	0.002

*Effect sizes of indirect effects of PTEs through post-migration stressors at different levels (at the mean and 1 SD above and below the mean) of the moderator resilience (CYRM-12), with bootstrapped confidence intervals (Boot CI)*.

**Figure 3:**
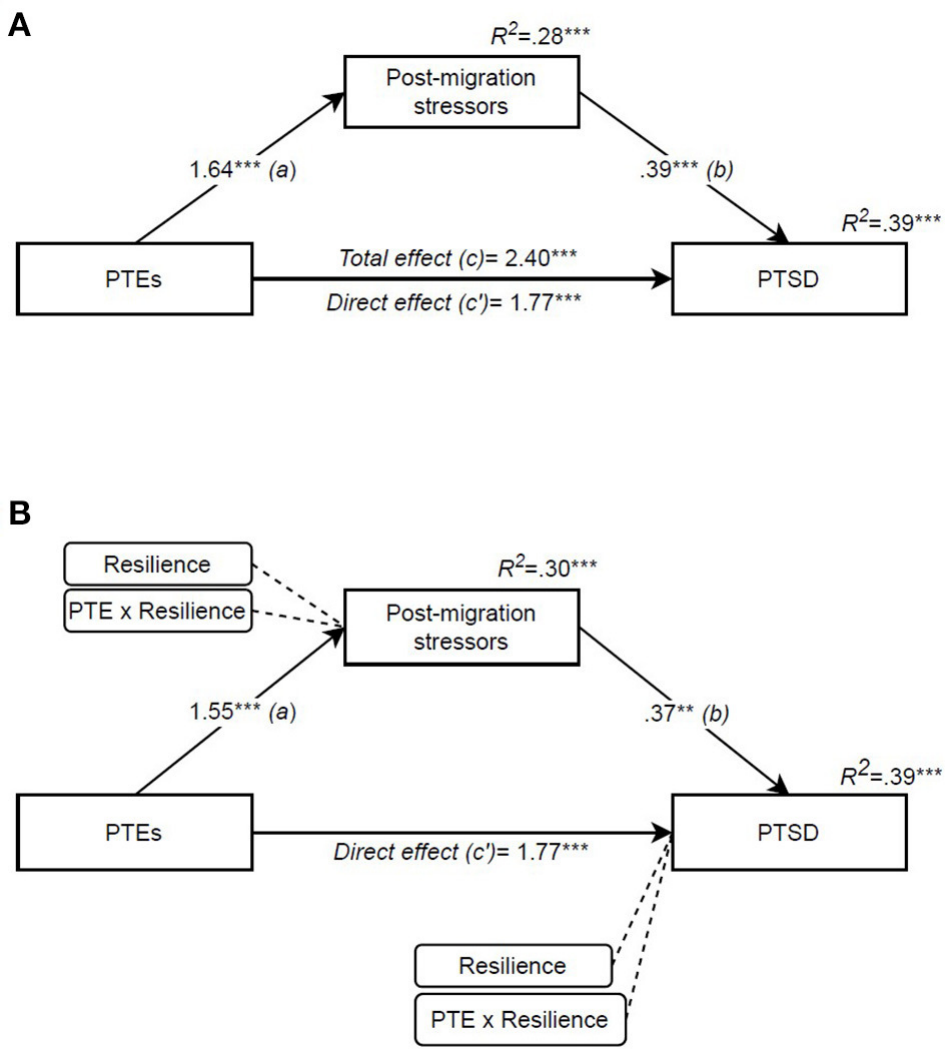
Potentially traumatic events' effect on PTSD mediated by post-migration stressors and moderated by resilience. **(A)** Mediation (PROCESS model 4), **(B)** Moderated mediation (PROCESS model 8). Independent variable: PTE [potentially traumatic events (RTHC)]. Mediator: post-migration stressors (RPMSS). Outcome: PTSD [post-traumatic stress disorder (CRIES-8)]. Moderator: resilience (CYRM-12). All coefficients are unstandardized. *p < 0.05, **p < 0.01, ***p < 0.001.

Repeating the same analyses for PTSD as an outcome showed that post-migration stressors mediated the effect of PTEs on PTSD, and that this was significant despite the direct effect (*c*′) also remaining significant [*indirect effect* (*a* and *b*): 0.63, 95% CI (0.23, 1.1); Figure 3A]. Adding resilience as a moderator (Figure 3B) did not result in significant changes.

**Figure 4:**
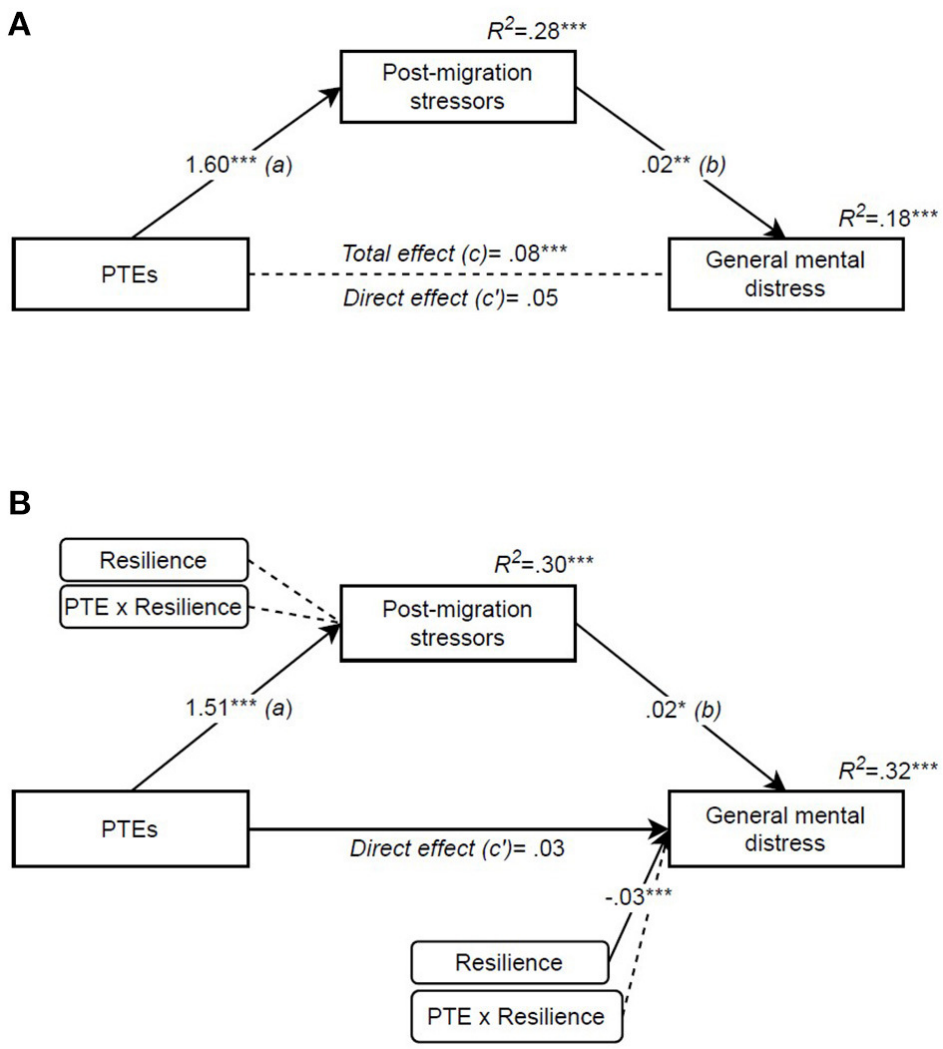
Potentially traumatic events' effect on general mental distress mediated by post-migration stressors and moderated by resilience. **(A)** Mediation (PROCESS model 4), **(B)** Moderated mediation (PROCESS model 8). Independent variable: PTE [potentially traumatic events (RTHC)]. Mediator: post-migration stressors (RPMSS). Outcome: general mental distress (HSCL-10). Moderator: resilience (CYRM-12). Covariate: gender. All coefficients are unstandardized. *p < 0.05, **p < 0.01, ***p < 0.001.

Lastly, the same analysis was conducted for general mental distress and, as shown in Figure 4A, the same mediating effect of post-migration stressors was found [*indirect effect* (*a* and *b*): 0.04, 95% CI (0.01, 0.07)]. The model accounted for 18% of the variance in distress scores [*F*_(3, 113)_ = 8.36, *p* < 0.001, *R*^2^ = 0.182], where gender contributed to about 5% of this variance (see Table 3). Adding resilience as a moderator (Figure 4B) did not result in significant interaction in the first stage of the model (*a*), nor the residual direct effect (*c*′); however, resilience directly contributed to the variance in general mental distress [total model: *F*_(5, 111)_ = 10.47, *p* < 0.001, *R*^2^ = 0.321]. Lastly, the indirect effect of PTEs was significant at all levels of resilience, and therefore resilience did not moderate the indirect effect. To summarise, the indirect effect of post-migration stressors was significant for all three outcomes, and this indirect effect was the same strength for all levels of resilience. Hence the index of moderated mediation was not significant (see Table 4).

A *post-hoc* correlational analysis with all resilience factors from the CYRM-12 scale was done to explore multidimensional aspects resilience, assessing differential impact by type of resilience factors, and not purely additive effects. As can be seen in Table 5, almost all items were positively correlated with HRQoL, and general mental distress showed very similar patterns but with negative correlations of somewhat lower values. Only four items were significant for PTSD.

**Table 5:** Correlations between items in CYRM-12 and HRQoL, PTSD, and general mental distress.

**CYRM-12 items**	**Mean**	**HRQoL**	**PTSD**	**General mental distress**	**Dimension**
I have people I look up to	3.23	0.365^***^	0.048	−0.106	Context
Getting an education is important to me	4.61	−0.018	0.056	−0.016	Context
My parents/caregivers know me well	4.03	0.453^***^	−0.235^**^	–0.371^***^	Caregiver
I try to finish what I start	3.91	0.180^*^	−0.056	−0.188^*^	Individual
I am able to solve problems without harming myself	4.11	0.195^*^	0.007	−0.118	Individual
I know where to go in my community to get help	3.72	0.452^***^	−0.171	–0.367^***^	Individual
I feel I belong at school	3.63	0.434^***^	−0.097	–0.281^***^	Context
My family stands by me in difficult times	4.28	0.407^***^	–0.345^***^	–0.287^***^	Caregiver
My friends stand by me in difficult times	3.69	0.501^***^	−0.020	–0.398^***^	Individual
I am treated fairly in my community	3.55	0.398^***^	–0.256*^*^	–0.440^***^	Context
I have opportunities to develop skills useful to me in later life	4.25	0.330^***^	–0.270*^*^	–0.251*^*^	Individual
I enjoy my family's/caregiver's cultural and family traditions	4.11	0.285^***^	−0.133	–0.234*^*^	Caregiver
CYRM-12 total	47.11	0.605^***^	−0.226^**^	–0.445^***^	

*Spearman's rho was used due to skewness and categorical values. Values significant after Bonferroni–Holmes correction are in bold.*

* *p < 0.05*,

** *p < 0.01*,

*** *p < 0.001. Green: Context resilience dimension. Orange: Caregiver resilience dimension. Blue: Individual resilience dimensions*.

## Discussion

Previous articles from the “Good Start” project (31, 37), have established the importance of direct and indirect pre- and post-migration risk factors on quality of life. This study adds to this knowledge by exploring the role of resilience factors, specifically promotive and protective mechanisms of influence. This also includes exploring outcomes separately, to distinguish between “positive” and “negative” pathways.

In this study, HRQoL was moderately good but lower than population norms (31, 45), similar to findings in other studies with refugees (51). Levels of general mental distress (36%) and PTSD (48%) were high, reflective of studies with Syrian youth resettled in neighbouring countries such as Jordan and Turkey (52, 53). However, Syrian youth in Sweden had much lower levels (34%) (54), suggesting a possible overestimation in this study. The participants reported high amounts of adverse experiences from war and flight akin to other studies of resettled Syrian children and youth (30, 52). The level of post-migration stressors was slightly lower compared both to Syrian adults (55) and other resettlement contexts, such as refugee camps and reception centres (36, 56). Nevertheless, they were a significant influence in all analyses. The resilience scores were skewed towards the positive end of the scale, and means were similar to Syrian and Jordanian youth in Jordan (22) but much higher than for example a group of Eritrean unaccompanied refugee minors (URM) in Sudan (57). However, making comparisons between different groups and contexts is difficult, as resilience is highly contextual and norm scores or cut-offs are less relevant (21).

Our findings support the presence of *promotive resilience* for HRQoL and general mental distress, supporting the first hypothesis of a direct main effect of resilience factors. However, no moderation effects were significant; as such, our second hypothesis of *protective resilience* buffering against negative effects was not supported. As proposed in the third hypothesis, the two risk factors (PTEs and post-migration stressors) created a mediational pathway where previous experiences increased post-migration stress, which in turn worsened outcomes. However, resilience did not moderate any part of the model, therefore not supporting the second part of the same hypothesis. A *post-hoc* correlation analysis indicated that relational and environmental factors were of particular importance, but also an additive effect of resilience factors.

### Promotive Resilience

*Promotive resilience* is described as resilience factors that directly improve healthy functioning despite—or independent of—exposure to risks (24–26). From this perspective, mental health can be viewed as a balance of “positives and negatives,” where positive resources increase healthier functioning whilst cumulative adversity impedes health. Descriptions of this mechanism often use scales as a metaphor, depicting the balancing act whereby individuals continually strive for homeostasis, and well-being is achieved when the scale is balanced (58). Our results found support for this mechanism, with a main effect explaining more than 20% of the variance in HRQoL and 12% in general mental distress. Some also describe this mechanism as compensatory or additive, similar to the results in Table 5 here, higher numbers of relevant items in the CYRM-12 scale increased explained variance. Seeing resilience as a balance of positives and negatives, differences between outcomes could be explained in terms of their levels: as HRQoL levels were only slightly reduced and a minority had high levels of general mental distress (negatives), the levels of resilience were enough to partly compensate (positives). However, since levels of PTSD were very high, higher levels of resilience might therefore be needed to compensate for these symptoms.

Several authors have tried to identify the driving force for the balance tipping towards resilience in contexts of risk; in this study, the relational and environmental factors seemed to be more important. However, socio-ecological models suggest that there is not one but several interactive resilience processes. In support of this, a review of resilience in children and youth affected by armed conflict found that participants perceived resilience to be a combination of personal strengths and supportive contexts (10). In this sense, individual *assets* and environmental *resources* are seen as independent contributors to mental health—both directly affecting feelings, competencies, and symptoms, but also interacting (12, 24, 25). For example, some individual coping mechanisms are not sustainable unless other environmental systems, such as family and school, support the adaptive behaviour (59, 60). A purely additive effect of separate resilience factors might therefore be too simplified, and both assets and resources need to be addressed in interventions. As such, the composite measure of CYRM-12 including individual, relational and contextual elements could be relevant, but the interactions between resilience factors need to be explored.

### Protective Resilience

The second resilience mechanism we explored was a potential moderating effect on the relationship between risks and outcomes, or *protective resilience*. This is described as resilience factors moderating or reducing the impact of a risk or stressor, with the metaphor of an umbrella shielding someone from the rain (25). This effect is found in several resilience studies (20), including those using CYRM (61, 62). However, we did not find support for this mechanism of resilience in any of our analyses. Other studies including refugee youth also show mixed results: some find that resilience moderates the relationship between risks and mental distress (63), whilst others do not (57, 64). The different results could be due to studies assessing different risk factors or measures of resilience. Or, that the *protective resilience* mechanism is relevant for some types of risk and resilience and not others (65). A lack of fit would then explain the absence of the buffering effects (66). Others suggest that the absence of *protective resilience* effects could be due to very high levels of current stress in the group—for example in detention, asylum or transit situations—as stress could overwhelm individuals or deplete their coping resources (66, 67). This result is also described in resilience theories, where risk factors can decrease the amount of resilience or inhibit it from having an effect (12). Our analyses also suggest that current post-migrations stressors had a large impact on all outcomes, even with levels being lower than in asylum or transit situations; moreover, results confirm their importance in worsening outcomes or delaying recovery (2, 33, 34, 37). Looking closely at effect sizes in Table 4, they appear to increase with higher resilience: this could be interpreted as the relationship between resilience and outcomes weakening with increasing amounts of PTEs. While this tendency should be explored further in future studies, it indicates that reducing stress may increase the resilience resources available to an individual (34).

Following this argument, it could be reasoned that *protective resilience* depends on the context of resettlement for refugees. Studies comparing refugee samples with other groups, such as majority youth, indicate that resilience shows different associations depending on the group. For example, type of coping (engagement or disengagement) has far greater impact on mental health in majority youth than in URM in Norway, and the interaction between daily hassles and disengagement coping is only a significant moderator for majority youth (14). Similarly, in a Dutch study, individual resilience moderated the negative effects of PTEs in majority youth but not in refugee youth (64).

### Differences Between Outcomes

With the notion that HRQoL and mental distress are different concepts and not opposing ends of a scale, each outcome was assessed separately. As in previous studies, we found that resilience correlated with higher HRQoL and less mental distress (52, 57). However, when controlling for risks, resilience was no longer associated with PTSD, a finding we share with other studies on Syrian youth (67, 68). The differences might have several explanations: for example, HRQoL and resilience are both socio-ecological constructs and may therefore share more variance, whilst PTSD symptomology is a narrower psychological construct. Another is that PTSD is more closely linked to past experiences, and HRQoL and general mental distress to more current stressors.

Some resilience factors were significant across all three outcomes. Two concerned the relationship with parents/caregivers, a third community acceptance (being treated fairly), and a fourth the opportunity for personal development. Interestingly, peer support and school belonging were very important for HRQoL and general mental distress, but not for PTSD: this might indicate that the traumatic experiences and symptoms are not easily shared between friends or at school. In this analysis, relational and contextual factors seemed more important for positive outcomes than individual assets. Lastly, getting an education was not significant for any of the outcomes, despite having a high mean score. This may indicate a ceiling effect, where education is inherently important to nearly all the participants and thus explains little variance. The considerable overlap between HRQoL and general mental distress—and the differences between types of mental distress—contradicts the idea that unique resilience pathways lead to “positive or negative” outcomes (10). However, it reiterates the importance of considering several relevant outcomes (28).

Another notable finding is that both mental distress and resilience were high in the group, which could support the notion that these co-exist (69). Studies have found that migrants and refugees have higher resilience than compared groups (22, 46). They also describe how positive growth and memories of trauma and hardship co-exist in their post-settlement narratives (70). This could be what some describe as resilient outcomes with loss (71), and indicate that individuals with PTSD are just as resilient as those without PTSD (72). Either way, it reiterates the importance of not labelling refugees as “vulnerable populations” and complicates evaluating resilience purely by outcome (69).

## Implications

One overall goal of exploring resilience is to enable the design and evaluation of evidence-based interventions or social policy which promote mental health. Our results suggest that promoting a range of resilience factors—both individual and environmental—could improve HRQoL and reduce general mental distress in refugee youth. However, a resilience focus must not distract attention from risks, nor obscure the suffering of children who are in need of clinical support. Interventions should address exposure to previous events and current stressors, as well as build individual capacities and provide support (73). Further, the lack of protective effects in this study does not mean that this mechanism is irrelevant in interventions, but rather that further studies are needed to clarify its role.

## Limitations

The cross-sectional design and interlinked nature of the variables under analysis prevent any assertion of causality or direction; moreover, self-report can overestimate the association between variables, as all measures are from the same source. Although the resilience measure was validated for this group and for face validity in this context, it may not contain other protective factors important for this context. The inclusion of a control group could have identified resilience factors of specific importance for the group, or determined whether direct main effects are unrelated to risk. Despite the power of calculations being sufficiently high, a larger sample size and strategic inclusion of individuals with more risk or lower resilience—such as the students who were absent from school—could have increased the likelihood of finding moderating effects. Although the sample used in our data was comparable to Syrians registered in Norway at the time, the generalizability of our findings to other countries or other groups of immigrant youth remains a question to be investigated in further research.

## Conclusion

In this study, the Syrian youth had high levels of resilience, as well as high levels of risk. The presence of *promotive resilience* suggests that broad interventions and policy targeted at whole groups would be beneficial for HRQoL and general mental distress, independent of risk factors or symptoms. These should aim to strengthen individual assets—such as coping mechanisms—and also build supportive environments in schools and families. However, reducing current stress and providing treatment for those in need could enable recovery and increase the efficacy of resilience interventions and already present resilience factors. No buffer effects—or *protective resilience*—were found, which could be due to high amounts of stressors inhibiting this mechanism, or that relevant resilience factors were not included in the resilience measure used. This should be explored in further studies.

The youth had high levels of PTSD and resilience factors had seemingly little influence on these symptoms. Instead, high resilience and symptomology seemed to co-exist, indicating that individuals with PTSD are just as resilient as those without.

## Data Availability Statement

The datasets used in the article will be made publicly available when the doctoral theses is complete and accepted.

## Ethics Statement

The study was reviewed and approved by Regional Research Ethics Committee of Norway East (Reference number 2018/192). Written informed consent to participate in this study was provided by the participants' legal guardian/next of kin.

## Author Contributions

All authors contributed to the study conception and design. Material preparation, data collection and analysis were performed by CD. The first draft of the manuscript was written by CD and all authors commented on previous versions of the manuscript. All authors have read and approved the final manuscript.

## Conflict of Interest

The authors declare that the research was conducted in the absence of any commercial or financial relationships that could be construed as a potential conflict of interest.

## Publisher's Note

All claims expressed in this article are solely those of the authors and do not necessarily represent those of their affiliated organizations, or those of the publisher, the editors and the reviewers. Any product that may be evaluated in this article, or claim that may be made by its manufacturer, is not guaranteed or endorsed by the publisher.
